# Unusual Course of Crystalline Keratopathy in a Patient with Graft-Versus-Host Disease

**DOI:** 10.4274/tjo.09582

**Published:** 2017-12-25

**Authors:** Başak Bostancı, Yonca Aydın Akova

**Affiliations:** 1 Okan University Faculty of Medicine, Department of Ophthalmology, İstanbul, Turkey; 2 Bayındır Hospital, Ophthalmology Clinic, Ankara, Turkey

**Keywords:** Crystalline keratopathy, Graft-versus-Host disease, fungal keratitis, dry eyes

## Abstract

We present a case of infectious crystalline keratopathy in a patient with Graft-versus-Host disease (GVHD) who developed satellite fungal keratitis. A 51-year-old man was referred for bilateral total persistent corneal epithelial defects with severe dry eye. Although persistent epithelial defect healed with medical therapy, he developed stromal keratitis with satellite lesions confirmed to be secondary to Candida albicans. After three months of antifungal treatment and debridement, improvement of the infiltrates was obtained. Crystalline keratopathy is an important clinical entity which may develop due to several causes. The microbial causes include not only bacteria but fungi as well. Careful investigation must be performed, especially for immune-compromised patients, in order to provide appropriate and timely treatment.

## INTRODUCTION

Crystalline keratopathy is a condition in which crystals are deposited in the anterior and/or mid-corneal stroma. Crystalline keratopathy of the cornea may be caused by several conditions including corneal dystrophies or systemic disorders, elevated serum immunoglobulins, corneal infections, or rejection of corneal grafts.^1,2,3^

Affected individuals present to ophthalmologists with symptoms of pain, redness, photophobia, and decreased vision. Clinical examination usually reveals conjunctival injection, chemosis, and corneal changes, which include branching crystalline opacities in the anterior/mid-corneal stroma associated with epitheliopathy.^4,5^

We present a unique case of a patient with graft-versus-host disease (GVHD) and crystalline keratopathy who developed keratitis with satellite lesions secondary to fungal keratitis, which regressed with antifungal treatment and corneal debridement.

## CASE REPORT

**Figure 1:**
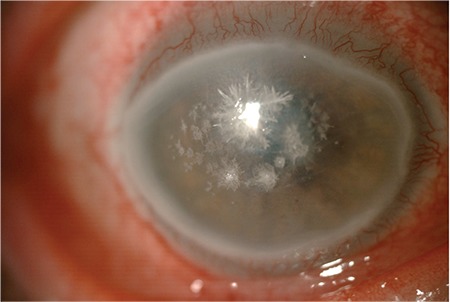
Slit-lamp examination of the right eye at the initial visit shows fine branching crystal deposits extending towards periphery in the anterior corneal stroma

A 51-year-old man was referred for evaluation of bilateral total corneal persistent epithelial defect (PED) with severe dry eye and multiple fine crystal deposits in the anterior corneal stroma of the right eye. He had a history of allogeneic bone marrow transplantation for acute myelocytic leukemia in 2011 and development of GVHD, which was diagnosed 2 months after transplantation. His best-corrected visual acuity (BCVA) was 20/400 in both eyes. Slit-lamp examination revealed total absence of corneal epithelium in both eyes and fine branching crystal deposits extending towards the periphery in the anterior corneal stroma of the right eye (Figure 1) and bilateral grade 3 nuclear cataract formation in both eyes. He had been treated with oral fluocortolone (40 mg/day), cyclosporine (150 mg/day), and sulfamethoxazole/trimethoprim (200 mg/day) and was using therapeutic contact lenses, prednisolone acetate eye drops twice daily, unpreserved artificial tears as needed, and moxifloxacin drops three times daily. The therapeutic contact lenses were removed. Conjunctival and corneal scrape samples were taken and yielded negative results in culture and cytology. Fortified vancomycin 50 mg/mL drops were given hourly for 3 days, then every two hours for 1 week. Observing a slow improvement in the epithelial defect and corneal infiltrates after 3 days, autologous serum eye drops (20% diluted with 0.9% sterile saline) four times daily and topical cyclosporine ophthalmic emulsion 0.05% (Restasis®) eye drops four times daily were added to the initial therapy and punctal occlusion was performed using silicone plugs (Punctal Plug F, FCI Ophthalmics). The patient was asked to come back for follow-up.

**Figure 2:**
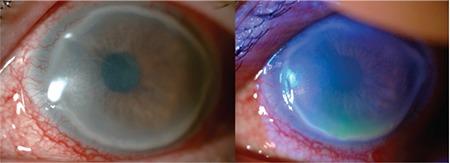
Small epithelial defect in the inferior cornea and mild edema in the left eye after treatment for persistent epithelial defect

During weekly follow-up, the epithelial defect got smaller in the right eye. A small epithelial defect in the inferior cornea and mild edema were observed in the left eye (Figure 2) after 2 weeks. His BCVA was 20/200 in the right and 20/200 in the left eye. Autologous serum eye drops were increased to six times a day. In addition, systemic doxycycline treatment (100 mg/day) for posterior blepharitis was prescribed. Fortified vancomycin therapy was ceased after 3 weeks.

**Figure 3:**
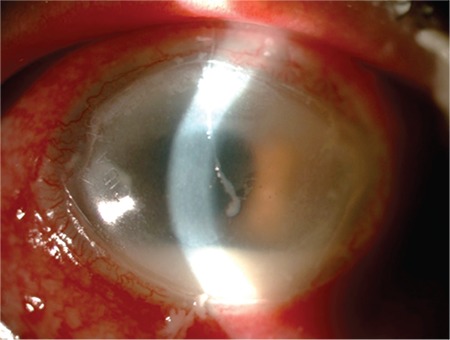
Hypopyon with keratitis in the left eye

He had uneventful weekly visits until he appeared with hypopyon and keratitis in the left eye 2 months later (Figure 3). Corneal scraping was performed and fortified antibiotic treatment was initiated hourly (cephazolin 50 mg/mL and vancomycin 50 mg/mL). Enterococcus spp. were isolated in cultures and shown to be sensitive to vancomycin in sensitivity testing. Therefore, the antibiotic therapy was sustained.

**Figure 4:**
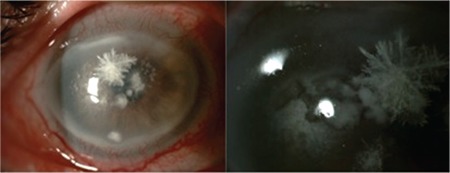
Newly formed peripheral and central stromal infiltrates in the right eye

**Figure 5:**
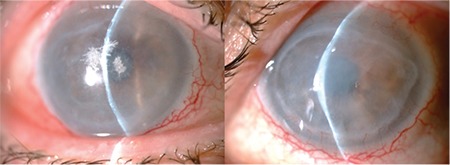
Both eyes after 6-week course of topical antifungal treatment

**Figure 6:**
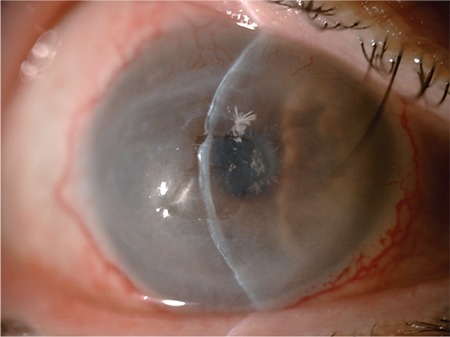
Right eye after debridement of the infiltrates

The hypopyon healed with topical fortified antibiotic regimen and systemic doxycycline therapy in 4 weeks. However, the patient then developed inferiorly localized peripheral and central stromal infiltrates adjacent to crystalline keratopathy in the right eye (Figure 4). Corneal scrapings were taken for diagnostic culture and cytology. The infection progressed and the patient developed new satellite stromal infiltrates in the central cornea of the right eye 3 days later. Corneal scrapings were taken again for diagnostic culture and cytology. Cytology showed aggregates of yeast elements in the corneal stroma and Candida albicans was identified as the causative organism in the culture. Topical 0.15% Amphotericin B and 1% voriconazole treatment was initiated hourly. After a 6-week course of topical antifungal treatment (Figure 5), the infiltrates were debrided with a diamond blade and a novel matrix regenerating agent (Cacicol 20®, polycarboxymethylglucose sulfate, Thea Labs) was prescribed to promote epithelial healing. After 12 weeks of treatment, the cornea healed with only slight irregularity (Figure 6). Antifungal therapy was stopped after epithelial healing was sustained in the 13th week. His final BCVA was counting fingers from 2 meters in both eyes secondary to mild stromal opacity and grade 3 nuclear cataracts, which was attributed to the systemic steroid treatment he was receiving for GVHD.

## DISCUSSION

GVHD is a devastating complication of allogeneic stem cell transplantation. The incidence of ocular GVHD is high after stem cell transplantation,^6^ and keratoconjunctivitis sicca and cicatricial conjunctivitis are two common ocular manifestations of this disease.^7^

Ocular involvement in GVHD appears as inflammatory destruction of the conjunctiva and lacrimal glands with fibrosis, decreased goblet cell density, and a resultant decrease in tear production.^8^ Major findings in the conjunctiva and cornea include punctate keratopathy, keratinization, epithelial thinning, and squamous metaplasia.^9^ Pseudomembranous pattern with corneal epithelial sloughing is generally considered an acute pattern of chronic ocular GVHD^10^, which was thought to be the reason for development of keratitis in our case since epithelial barrier function was impaired.

In the presence of certain risk factors, such as corneal hypoesthesia, diabetic keratopathy, limbal stem cell deficiency, dry eye disease, and certain keratopathies, epithelial defects can persist despite standard therapies. When a patient shows no response to treatment after approximately two weeks, they are said to have a PED.^11^ Aggressive lubrication, bandage soft and scleral contact lenses, pressure patching, autologous serum, punctal occlusion, debridement, amniotic membrane grafting, and limbal stem cell transplantation are some of the treatment options for PED.^12^ In our case, punctal occlusion, aggressive lubrication and autologous serum eye drops were used, as well as a novel matrix regenerating agent (Cacicol 20®, polycarboxymethylglucose sulfate, Thea Lab). Cacicol, which is a structural analogue of glycosaminoglycans, mimics heparin sulfate (HS) and is thought to replace the degraded HS and produce a suitable environment for recruiting growth factors necessary for corneal repair, especially in eyes with PED.^13^

Cases of crystalline keratopathy secondary to fungi have been presented in the literature.^14^ In the present case, the crystalline keratopathy developed in a GVHD patient resolved after antifungal therapy, which indicates that the causative agent might have been fungi. Infectious crystalline keratopathy may arise de novo or after surgical procedures such as refractive surgery or penetrating keratoplasty.^4,5^ Streptococcus viridans is the most common organism to cause crystal deposits followed by Staphylococcus epidermidis, Streptococcus pneumonia, Haemophilus spp., and enterococci.^15^ However, Candida spp. and atypical organisms such as mycobacteria must be kept in mind, especially in immune-suppressed conditions such as patients who use chronic corticosteroids or abuse topical anesthetic eye drops.^14^

When the clinical course of our patient was reviewed, it could not be ascertained whether the crystalline keratopathy initially observed was secondary to fungal infection. However, the size and number of crystals in crystalline keratopathy regression strongly suggests a fungal etiology. On the other hand, our patient had been immune-compromised and he had epithelial irregularity, PED, and severe dry eye secondary to GVHD, which may have an impact on the development of corneal infection. To draw a conclusion, we believe that ophthalmologists must be ready for various clinical courses in a patient with GVHD and that fungal and opportunistic pathogens must be kept in mind while dealing with infections, especially when epithelial integrity is lost.
